# Supplementary material for: Heterostructured α-Bi2O3/BiOCl Nanosheet for Photocatalytic Applications
Source: Nanomaterials (Basel). 2022 Oct 16;12(20):3631. doi: 10.3390/nano12203631 (PMC9608947; doi:10.3390/nano12203631)
Supplement: Supplementary file 1 [file nanomaterials-12-03631-s001.zip › nanomaterials-1965565-supplementary.pdf]

nanomaterials

Supplementary Materials

Heterostructured α-Bi_2O_3/BiOCl Nanosheet for Photocatalytic Applications

Daoguang Teng [1,†], Jie Qu [1,†], Peng Li [1], Peng Jin [1], Jie Zhang [2], Ying Zhang [1,*] and Yijun Cao [1,*]

[1] School of Chemical Engineering and Zhongyuan Critical Metals Laboratory, Zhengzhou University, Zhengzhou 450001, China
[2] School of Ecology and Environment, Zhengzhou University, Zhengzhou 450001, China
* Correspondence: zhangying777@zzu.edu.cn (Y.Z.); yijuncao@zzu.edu.cn (Y.C.)
† These authors contributed equally to the work.

Calculation of band gap energy E_g:

$$\alpha hv = A(hv - E_g)^{n/2} \tag{S1}$$

where α, h, v, and A are the optical absorption coefficient, Planck constant, photon frequency, and proportional constant, respectively. Coefficient n closely relies on the characteristics of semiconductor: the value of direct bandgap semiconductor (Bi_2O_3) was 1, while the value of indirect bandgap semiconductor (BiOCl) was 4. The E_g of Bi_2O_3 and BiOCl was gained by plotting energy (hv) by $(\alpha hv)^2$ and $(\alpha hv)^{1/2}$, respectively; and extending the obtained straight-line segment of the graphic to x-axis (Figure 4b,c).

Calculation of valence band edge E_{VB} and conduction band edge E_{CB}:

$$E_{CB} = X - E_C - 0.5E_g \tag{S2}$$

$$E_{VB} = E_{CB} + E_g \tag{S3}$$

In this formula, X, E_{CB} and E_{VB} are the absolute electronegativity of the semiconductor (the geometric mean of the constituent atoms), the position of the conduction band edge and the position of the conduction valence band, respectively. The X values of Bi_2O_3 and BiOCl were 5.95 and 5.00 eV, while the calculated E_g of Bi_2O_3 and BiOCl were 2.85 and 3.52 eV, respectively. Therefore, E_{VB} of Bi_2O_3 and BiOCl were 2.88 and 2.26 eV, while E_{CB} of Bi_2O_3 and BiOCl were 0.03 and −1.26 eV.

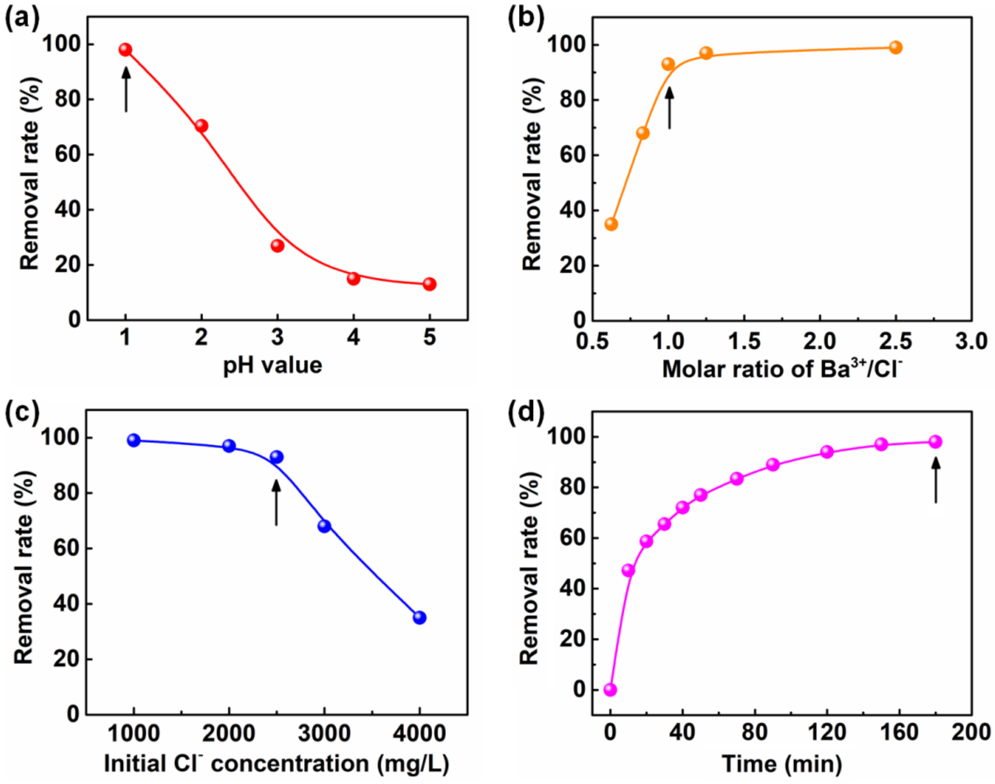

Figure S1. Removal rate of Cl^- onto Bi_2O_3 under different conditions: (**a**) pH value, (**b**) molar ratio of Bi^{3+}:Cl^-, (**c**) initial Cl^- concentration, and (**d**) contact time.

As seen in Figure S1a, the Cl^- removal rate increased with the reduction of pH value. At pH = 1, the removal rate reached the maximum value of >99%. From Figure S1b, with the molar ratio of Bi^{3+}:Cl^- increased from 0.625:1 to 1:1, the Cl^- removal rate increased from 35% to 95%, and there was no obvious increasement under higher molar ratio (1.25:1 and 2.5:1). While the Cl^- removal rate decreased with the increasement of initial Cl^- concentrations (**Figure S1c**), and the initial concentration of 2500 mg/L was selected due to the high utilization efficiency of Bi^{3+}. With the prolonging of contact time, the Cl^- removal rate gradually increased (Figure S1d). Hence, the optimal conditions for Bi_2O_3 to remove Cl^- were pH = 1, Cl^- concentration = 2500 mg/L, Bi^{3+}:Cl^- ratio = 1:1, and contact time = 180 min.

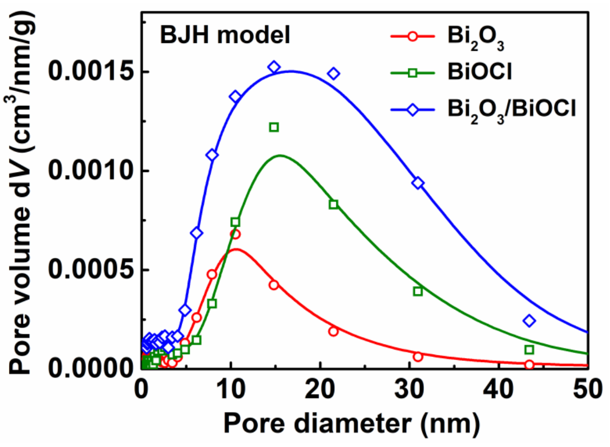

Figure S2. The Barrett–Joyner–Halenda (BJH) pore size distributions of three samples.

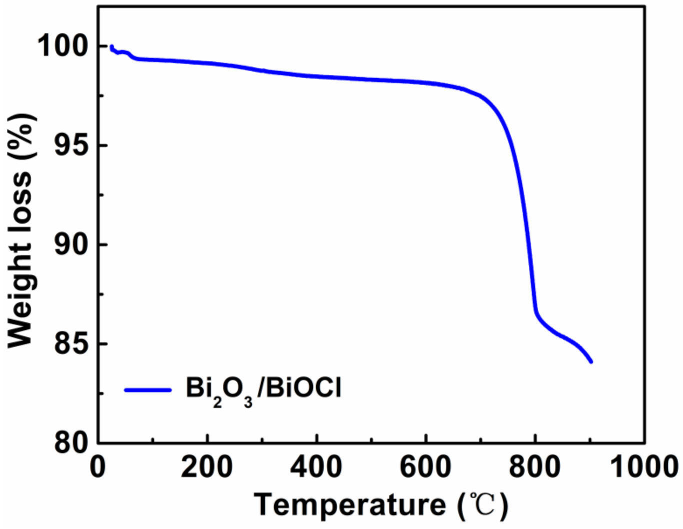

Figure S3. The TG curve of Bi_2O_3/BiOCl in nitrogen atmosphere.

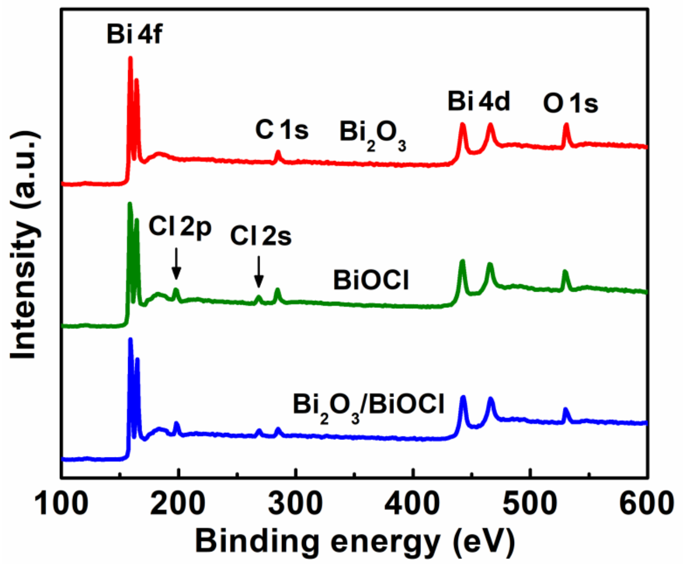

Figure S4. The XPS survey spectra of Bi_2O_3, BiOCl and Bi_2O_3/BiOCl.

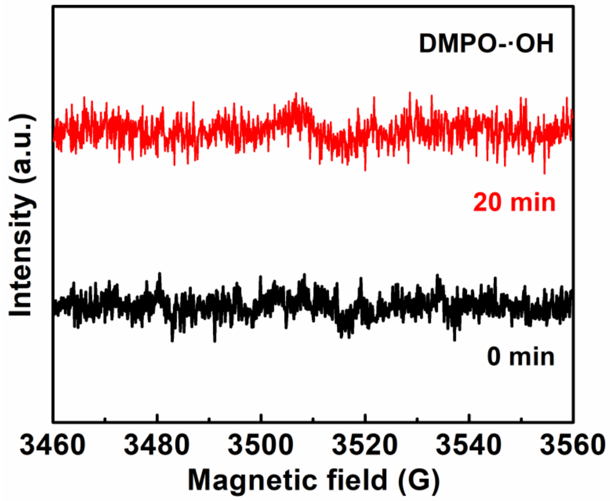

Figure S5. The electron spin resonance (ESR) spectra by adding 5,5-dimethyl-1-pyrroline N-oxide (DMPO) to capture •OH.

Table S1. Pore texture parameters of three samples.

Samples	S_{BET} (m^2/g)	V_t (cm^3/g)	d_a (nm)
Bi_2O_3	2.3	0.009	15.7
BiOCl	6.8	0.031	18.2
Bi_2O_3/BiOCl	11.2	0.058	20.7

Remark: S_{BET} — the specific surface area using the BET method,
V_t — the total pore volume at relative pressure P/P_0 of 0.99,
d_a — average pore diameter was calculated as: d_a=4000V_t/S_{BET}.

Table S2. Optical properties of Bi_2O_3 and BiOCl.

Material	Absorption edges (nm)	E_g (eV)	E_{VB} (eV)	E_{CB} (eV)
Bi_2O_3	440	2.85	2.88	0.03
BiOCl	380	3.52	2.26	−1.26

Remark: E_g — the band gap energy of material,
E_{VB} — the valence band edge of material,
E_{CB} — the conduction band edge of material.
